# Factors influencing older adults’ acceptance of voice assistants

**DOI:** 10.3389/fpsyg.2024.1376207

**Published:** 2024-03-07

**Authors:** Xiancai Cao, Hao Zhang, Bolin Zhou, Dahua Wang, Chenhong Cui, Xuejun Bai

**Affiliations:** ^1^Key Research Base of Humanities and Social Sciences of the Ministry of Education, Academy of Psychology and Behavior, Tianjin Normal University, Tianjin, China; ^2^Faculty of Psychology, Tianjin Normal University, Tianjin, China; ^3^Tianjin Social Science Laboratory of Students' Mental Development and Learning, Tianjin, China; ^4^School of Management, Tianjin Normal University, Tianjin, China; ^5^Institute of Developmental Psychology, Beijing Normal University, Beijing, China

**Keywords:** older adults, voice assistants, technology acceptance model, behavior intention, dispositional resistance to change

## Abstract

**Introduction:**

Voice assistants (VAs) have the potential to uphold and enhance the quality of life for older adults. However, the extent to which older adults accept and benefit from VAs may be relatively modest.

**Methods:**

This study developed a comprehensive model combined with product and personal characteristics to explain the acceptance of VAs among older adults, using semi-structured interviews (Study 1) and questionnaires (Study 2).

**Results:**

Results revealed that in terms of product characteristics, perceived usefulness and perceived enjoyment significantly affect behavior intention. Regarding personal characteristics of older adults, technological self-efficacy and dispositional resistance to change significantly affect behavior intention. However, no direct impact of perceived ease of use and perceived trust on behavior intention. Additionally, perceived enjoyment influenced both perceived ease of use and perceived usefulness.

**Discussion:**

Results suggested the significant role of technology self-efficacy and dispositional resistance to change in predicting the acceptance of VAs among older adults. Our newly developed model offers valuable insights for tailoring VAs to this demographic during design and implementation.

## 1 Introduction

Voice interaction is highly effective and humanized in Human-Computer Interaction (HCI) (Turow, 2021; Calahorra-Candao and Hoyos, 2024). The rapid development of intelligent voice technology, facilitated by advancements in deep learning, Internet of Things (IoT) technology, and enhanced computing power, is revolutionizing societal norms (Pham Thi and Duong, 2022; Ossadnik et al., 2023). A prominent manifestation of this technology is found in voice assistants (VAs). According to Juniper Research (2021), the number of VAs is projected to exceed the global population, reaching 8.4 billion by 2024.

### 1.1 Voice assistants and older adults

According to the 2022 Revision of World Population Prospects (Department of Economic and Social Affairs of United Nations, 2022), the proportion of the global population aged 65 and above is projected to increase significantly, exceeding 1.5 billion by 2050. As of the end of 2022, the elderly population aged 60 and above in China reached 280.04 million (National Bureau of Statistics of China, 2023). The current study defined elderly as the people aged 60 and above. As VAs become increasingly popular and permeate into our daily lives, it is foreseeable that there will be an increasing number of elderly users using VAs in the future.

Previous research has indicated that VAs can support older people in various aspects. Firstly, due to the significant decline in cognitive and physical functions caused by aging, the ability of older adults to perform instrumental activities of daily living (IADLs), such as using smartphones and computers and engaging in shopping, is weakened (Lawton and Brody, 1969; Liu et al., 2023; Werner et al., 2023). Voice interaction offers a more natural and straightforward interaction, making it easier for older people to learn and operate (Kebede et al., 2022; Kuoppamäki et al., 2023). Additionally, VAs can facilitate communication between older adults and others, especially those with visual or hand function impairments (Kowalski et al., 2019). It also found that VAs can serve as social companions to some extent (Corbett et al., 2021; Yan et al., 2024). Lastly, VAs can also assist in daily health activities such as health tracking, medication management, and meal planning, which could support the elderly’s daily lives (Nallam et al., 2020; Schlomann et al., 2021; Yan et al., 2024).

Despite the potential of VAs to support the lives of the elderly and their overall good usability, previous research has indicated that the willingness of older adults to adopt VAs is relatively low (Song et al., 2022). Additionally, VAs were initially designed for younger people, and the designers are usually younger, having grown up in a more technologically advanced environment (Kim and Choudhury, 2021). As a result, the product design often overlooks older adults’ physiological and psychological characteristics. Therefore, it is crucial to identify the factors that promote or hinder the use of VAs among older adults.

## 2 Literature review

To investigate the factors influencing the acceptance of VAs among older adults, we will review three aspects: the theoretical models related to technology acceptance, factors influencing technology acceptance among older adults, and research on the acceptance of VAs among older adults.

### 2.1 The theoretical models related to technology acceptance

Several theories have been proposed to understand the factors driving user technology acceptance, including the Theory of Reasoned Action (TRA, Fishbein and Ajzen, 1975), the Technology Acceptance Model (TAM, Davis, 1989), the Technology Acceptance Model 2 (TAM2, Venkatesh and Davis, 2000), and the Unified Theory of Acceptance and Use of Technology (UTAUT, Venkatesh et al., 2003). Among these proposed technology acceptance models, TAM is currently the most widely used model for predicting technology acceptance (Ma et al., 2021; Yu et al., 2022). TAM was initially proposed to provide an explanatory framework for the factors influencing computer systems within organizational settings. As TAM theory evolved, it has been widely applied to investigate user acceptance of new technologies (Nagle and Schmidt, 2012; Zhao et al., 2018; Zhu and Cheng, 2022). Perceived usefulness and perceived ease of use, as proposed by TAM, are the main determinants influencing users’ intention of technology acceptance behavior. Perceived ease of use refers to the user’s subjective perception of the ease or difficulty of utilizing a particular technology. Perceived usefulness refers to the user’s subjective perception of the degree to which a technology is beneficial during use. Moreover, perceived ease of use positively influences perceived usefulness.

Furthermore, Ram and Sheth (1989) proposed the innovation resistance theory (IRT) based on consumers’ resistance to innovation, which categorized innovation resistance into functional barriers and psychological barriers. Functional barriers encompass risks, value, and usage barriers, while psychological barriers include traditional and image barriers. Several studies employ the IRT theory to analyze consumer innovation adoption behavior, especially in the initial adoption stage (Borraz-Mora et al., 2017; Dotzauer and Haiss, 2017; Leong et al., 2021). IRT has emerged as the favored framework among scholars specializing in the study of innovation resistance, either as a standalone model (Borraz-Mora et al., 2017; Hew et al., 2019) or when integrated with other established frameworks such as UTAUT (Lian and Yen, 2014) and TAM (Agag and El-Masry, 2016).

### 2.2 Application of technology acceptance-related theories to older adults

Given older adults’ physiological and psychological characteristics, many studies incorporate personal and perceptual characteristics variables when exploring technology acceptance among older adults using relevant theoretical frameworks. For instance, some studies have indicated that technological self-efficacy and technology anxiety play essential roles in the technology acceptance of older adults (Peral-Peral et al., 2020; Jeng et al., 2022). Chen and Chan (2014) found that these constructs may better predict technology acceptance behavior of older adults than the conventionally attitudinal factors (usefulness and ease of use). Furthermore, older adults tend to have a slower processing speed and a more challenging learning process when accepting and learning new technologies (Kebede et al., 2022). If using technology is enjoyable, older adults are more likely to adopt it (Talukder et al., 2020). Studies have found that incorporating perceived enjoyment can improve the predictive power of technology acceptance-related models (Phibbs, 2021; Zeng and Chen, 2022). Additionally, the fear of technology intrusiveness among older adults has been repeatedly identified as a significant barrier to adopting technologies (Fischl et al., 2017; Kong and Woods, 2018; Zambianchi et al., 2019). Song et al. (2022) found that perceived trust directly impacts the acceptance of VAs.

On the other hand, dispositional resistance to change (DRTC) within personality traits is also considered a valid antecedent for the technological acceptance of older adults. Due to the pro-innovation bias (Talke and Heidenreich, 2014), most researchers believe that consumers are willing to change and are thus tempted to try innovative products, as long as the benefits of the technology, such as perceived usefulness and perceived ease of use, are emphasized. However, the reality often is that consumers tend to reject innovation without considering the potential of the product, leading to the adoption process ending before it even begins. This is particularly evident among older adults (Ma et al., 2021). Despite successful implementation of innovation, there may still be resistance (Laukkanen et al., 2008). Consumers often resist innovation due to changes in their current habits or norms required for accepting new ideas (Snyder, 1961). Previous research on VAs has found that older adults accustomed to traditional interaction methods involving input and output devices tend to exhibit resistance when adopting VAs (Trajkova and Martin-Hammond, 2020). However, no study has delved into the influence of DRTC on behavior intention of VAs among older adults. Furthermore, despite the inherent simplicity of VA interactions, older adults still encounter misunderstandings and challenges when using VAs during the initial stages (Kim, 2021), which may lead older adults with higher levels of DRTC to discontinue usage altogether.

### 2.3 Research on the acceptance of voice assistants among older adults

In recent years, the prospects for applying for VAs have expanded extensively. Numerous studies have initiated exploring the adoption and utilization of VAs among older adults.

Currently, research primarily focused on the advantages and disadvantages of VAs. VAs provided simplicity, convenience, and easy accessibility. It could support and enhance the social engagement, autonomy, and leisurely activities of older adults (Kowalski et al., 2019; Pradhan et al., 2020; Kim, 2021; Song et al., 2022; Werner et al., 2023). However, within VAs utilization, certain predicaments came to the forefront. Concerns about the privacy and security of financial information and recorded dialogues emerged (Bonilla and Martin-Hammond, 2020; Pradhan et al., 2020; Kim, 2021; Song et al., 2022; Werner et al., 2023). Additionally, challenges related to voice recognition were documented, including instances of speech misrecognition, difficulties in recalling specific commands, and issues with device response timing (Kowalski et al., 2019; Pradhan et al., 2020; Kim, 2021; Werner et al., 2023). Some older adults also believed VAs had not exhibited significant advantages and failed to demonstrate any discernible utility (Trajkova and Martin-Hammond, 2020; Kim, 2021).

Further research has delved into the impact of personal characteristics of older adults on the acceptance of VAs. Trajkova and Martin-Hammond (2020) found that many older adults refrained from using Echo. This avoidance was rooted in their belief in their ability to complete tasks independently. They hold a deep appreciation for this autonomy, potentially earmarking the adoption of such technology for a period when their physical state experiences a decline. Moreover, a lack of pertinent knowledge regarding VAs among the elderly may also engender difficulties in their utilization (Pradhan et al., 2020; Kim, 2021; Werner et al., 2023). Lastly, Song et al. (2022) have posited that self-efficacy might indirectly influence the acceptance of VAs among the elderly.

## 3 The current study

Previous studies have demonstrated the factors that promote or hinder older adults’ acceptance of VAs, primarily focusing on the characteristics of VA products, as well as older adults’ knowledge, experience, and physical condition as personal characteristics. DRTC and technological self-efficacy may have a stronger impact on older adults’ acceptance of technology (Chen and Chan, 2014; Touchaei and Hashim, 2023; Werner et al., 2023), but have been overlooked in the investigation of VAs’ acceptance. Therefore, it is necessary to further explore whether there are additional potential factors influencing the acceptance of VAs among older adults.

Moreover, to our understanding, most studies on the acceptance of VAs by older adults have relied on a single method. Except for Song et al. (2022), who used a questionnaire, current research has predominantly used qualitative methods to understand the viewpoints of the elderly towards VAs (Kowalski et al., 2019; Pradhan et al., 2020; Kim, 2021; Werner et al., 2023). However, the qualitative method has limitations, such as subjectivity, small sample sizes, and the inability to explore relationships between variables thoroughly. Further quantitative methods based on qualitative methods are an effective approach to remedy this limitation. Moreover, cross-validation between these two methods can enhance the credibility and accuracy of the research results, thereby bolstering the study’s robustness and comprehensiveness.

Besides, COVID-19 may have also altered the attitudes of older adults towards digital technology. While previous research has indicated that privacy concerns can hinder the adoption of VAs among older adults (Bonilla and Martin-Hammond, 2020; Pradhan et al., 2020; Kim, 2021; Song et al., 2022; Werner et al., 2023), the ongoing stress caused by the pandemic has highlighted the potential of VAs to support independent living and help older adults cope with the pressures brought by COVID-19 (Carstensen et al., 2020; Werner et al., 2023). This may lead older adults to prioritize the benefits of adopting VAs over privacy concerns, a trade-off that could extend into the post-COVID-19 era. Furthermore, factors such as perceived ease of use and self-efficacy in using VAs have been found to play a crucial role in the acceptance of VAs by older adults (Song et al., 2022). However, the COVID-19 pandemic has accelerated the adoption of digital technology among older adults (Diehl et al., 2022), potentially impacting the influence of perceived ease of use and self-efficacy on behavioral intentions regarding VAs adoption (Kim and Choudhury, 2021). Therefore, it is necessary to re-examine the impact of these factors on the acceptance of VAs.

**Figure 1:**
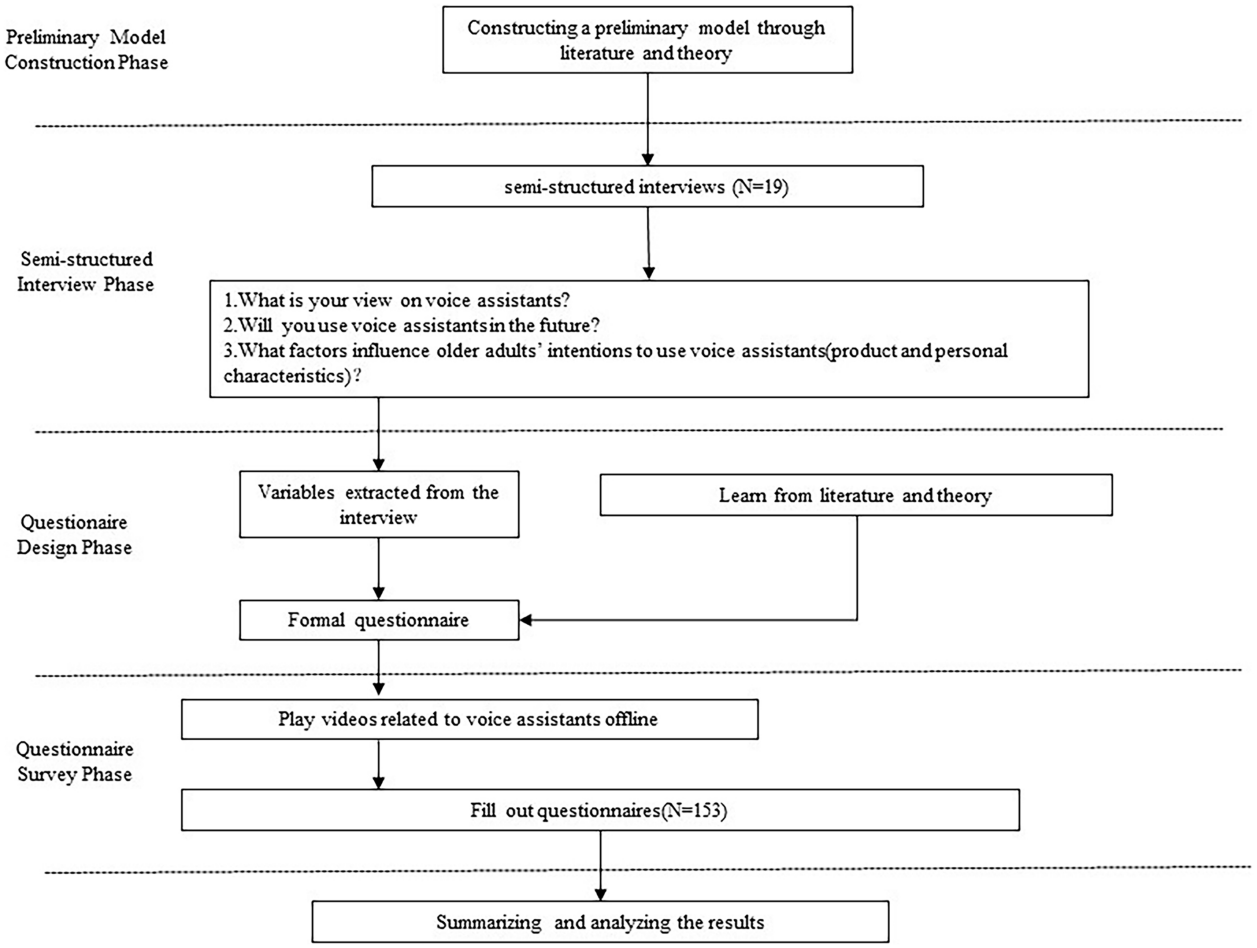
Research process of current study.

To address these research gaps, we propose the following approach. First, we will extract factors influencing the acceptance of VAs among older adults from existing literature and theories and develop a preliminary model. Second, we will conduct semi-structured interviews to explore further the factors influencing the acceptance of VAs among older adults. Lastly, we will incorporate the newly emerged factors from the interviews into the preliminary model and employ partial least squares structural equation modeling (PLS-SEM) to determine the mechanisms influencing the acceptance of VAs among older adults. The research process is illustrated in Figure 1.

## 4 Preliminary hypothesis development

Based on existing literature, we propose a preliminary model that extends TAM with the addition of relevant constructs specific to VAs (i.e., perceived enjoyment, value barrier, and perceived trust) (Nguyen et al., 2018; Trajkova and Martin-Hammond, 2020) and older adults (i.e., technological self-efficacy) (Chen and Chan, 2014).

Perceived trust and self-efficacy are unique characteristics of VAs and have been widely used as standard metrics for measuring experience in VAs research (Kim and Choudhury, 2021; Song et al., 2022). Value barrier also has been repeatedly mentioned in interview-based studies on older adults’ perspectives on VAs (Trajkova and Martin-Hammond, 2020; Kim and Choudhury, 2021). Perceived enjoyment is also a relevant and unique factor for VAs, and has been explored among younger age groups (Nguyen et al., 2018).

### 4.1 Perceived usefulness, perceived ease of use, and behavioral intention

Perceived usefulness and perceived ease of use are determinants in TAM, widely used in exploring technology acceptance among older adults. Meta-analyses have shown positive effects of perceived usefulness and perceived ease of use on behavioral intention (Zhou et al., 2020; Ma et al., 2021). Previous studies have extensively considered the influence of perceived usefulness and perceived ease of use on VAs acceptance (Nguyen et al., 2018; Song et al., 2022; Zhong et al., 2022). Given the significant advantages of VAs compared to other technologies, such as natural and simple interactions that can support independent living among older adults (Song et al., 2022), we expect that the practical benefits and convenience will positively influence older adults’ behavioral intention to use VAs. Furthermore, the similarity between interactions with VAs and interpersonal dialogues in daily life enables older adults to learn intuitively, and effortless learning can further enhance users’ perceived usefulness. Previous research has already demonstrated the positive relationship between perceived ease of use and perceived usefulness in the context of VAs (Nguyen et al., 2018; Song et al., 2022). Therefore, the following hypotheses are proposed:

*H1*: Perceived usefulness positively affects behavioral intention.

*H2*: Perceived ease of use positively affects behavioral intention.

*H3*: Perceived ease of use positively affects perceived usefulness.

### 4.2 Perceived enjoyment

Perceived enjoyment refers to the extent to which older adults perceive using VAs as entertaining and delightful, besides any expected performance outcomes (Davis et al., 1992; Nguyen et al., 2018). Empirical evidence from the research indicates that PE is one of the main reasons why mobile users access VAs (Nguyen et al., 2018). Hassenzahl (2018) also suggests that products should consider both utilitarian value and hedonic value, as the combination of these values better reflects the beneficial characteristics of the product. Therefore, in addition to perceived usefulness and perceived ease of use, Perceived enjoyment becomes another significant driving factor explaining behavioral intention in our comprehensive model. We propose the following hypothesis:

*H4*: Perceived enjoyment positively influences behavioral intention.

### 4.3 Value barrier

According to Ram and Sheth (1989), in this study, value barrier refers to the evaluation of value that older adults assign to VAs and their alternatives. Consumers typically use their current products as reference points. Suppose a new product does not offer more excellent value than the reference point, in that case, they are less likely to consider switching to an alternative because the perceived drawbacks of changing from the existing norm seem to outweigh the benefits (Leong et al., 2021). Trajkova and Martin-Hammond (2020) found that the main reason older adults discontinued using VAs was their difficulty finding valuable uses. Several studies have indicated that value barrier had a negative impact on behavioral intention in various contexts, including online shopping (Lian and Yen, 2014), mobile commerce (Moorthy et al., 2017), mobile banking (Laukkanen, 2016), and mobile payment systems (Kaur et al., 2020). Furthermore, when consumers attempt to assess the value difference between innovative and existing products, they consider various alternatives to accomplish their tasks, and the functions of VAs can also be replaced by other mediums (Trajkova and Martin-Hammond, 2020). This may also affect the perceived usefulness of VAs. Therefore, we propose the following hypotheses:

*H5*: Value barrier negatively influences behavioral intention.

*H6*: Value barrier negatively influences perceived usefulness.

### 4.4 Perceived trust

Trust is a significant factor influencing users’ adoption of technology. Security concerns, privacy risks, and distrust are common reasons for digitally disengaging, mainly in web-based digital technologies (Kebede et al., 2022). VAs must record users’ voice commands and daily speech to respond effectively, which may lead users to perceive more risks than other technologies (Song et al., 2022). In this case, trust helps alleviate users’ concerns about sharing and potentially misusing their personal information (Nguyen et al., 2018). Therefore, we propose the following hypothesis:

*H7*: Perceived trust positively influences behavioral intention.

### 4.5 Technological self-efficacy

According to Chen and Chan (2014), in this study, technological self-efficacy refers to the belief of older adults in their ability to use VAs successfully. Contrary to a widespread belief or marketing claim that interacting with VAs is effortless due to their conversational capabilities, most participants found it challenging to engage in a smooth conversation with the technology (Kim, 2021). Furthermore, older adults face additional challenges in using VAs due to their lack of similar technological upbringing as younger generations (Charness and Boot, 2022) and age-related declines in physiological and cognitive functions (Kim, 2021).

The social cognitive theory posits that a heightened level of self-efficacy can enhance cognitive processes (Bandura, 1995). For older adults, a strong sense of self-efficacy can help them maintain a positive and proactive outlook. When faced with challenges in using VAs, old adults with high self-efficacy are more likely to approach the situation optimistically and proactively rather than dwelling on the difficulties. Werner et al. (2023) found that older adults who proactively initiate the use of VAs may believe in their ability to interact successfully with VAs, while those who do not initiate such usage may lack the same level of confidence. Numerous studies have also revealed a direct positive correlation between self-efficacy, perceived ease of use, and behavioral intention (Song et al., 2022; Zhu and Cheng, 2022). Therefore, we propose the following hypotheses:

*H8*: Technological self-efficacy positively influences perceived ease of use.

*H9*: Technological self-efficacy positively influences behavioral intention.

**Figure 2:**
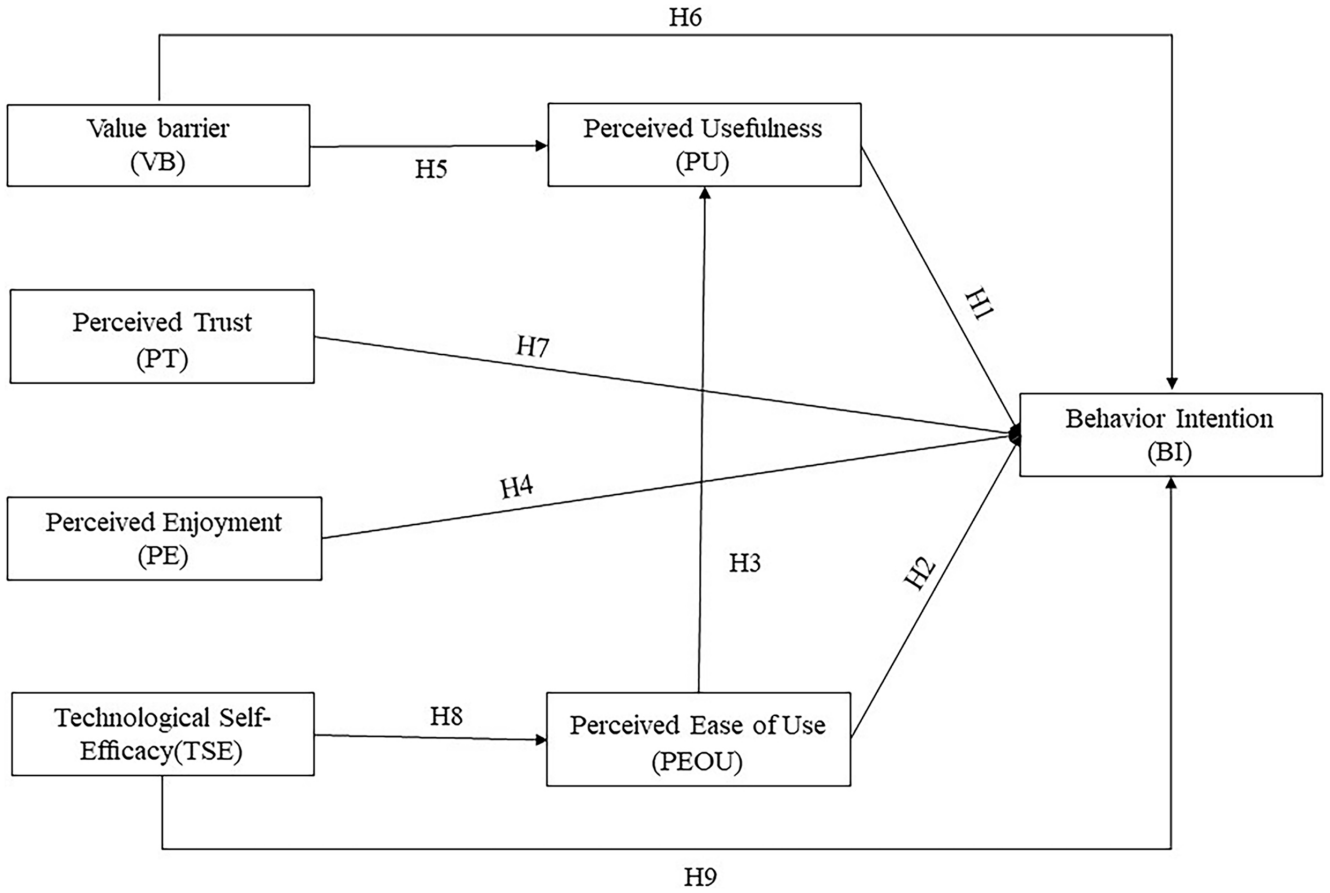
The preliminary model of current study.

Based on the above, the preliminary research model is illustrated in Figure 2.

## 5 Study 1: semi-structured interviews

The aim of the semi-structured interviews was to explore further the factors influencing the acceptance of VAs among older adults and validate the preliminary model.

### 5.1 Participants

For practicality and accessibility, we collaborated with Tianjin communities using convenient sampling to recruit participants. A total of 19 participants (10 females), with a mean age of 71.68 and an age range of 61–80, were recruited to participate in the semi-structured interviews. All older adults signed an informed consent form and completed the Mini-Mental State Examination (MMSE) cognitive test (Folstein et al., 1975). The total score range of the MMSE scale is 0 to 30 points. With test scores influenced by cultural background, normal cutoff values are as follows: for illiterate individuals, the cutoff is >17 points; for those with primary education, the cutoff is >20 points; and for those with junior high school education or higher, the cutoff is >24 points. The participants were selected based on three criteria: normal cognition, age over 60 years, and experience using smart devices such as smartphones, tablets, smartwatches, and smart speakers. We aimed to include both current and potential users of VAs, and prior experience with smart devices was a prerequisite for using VAs (Song et al., 2022).

### 5.2 Procedure

Considering that older adults may be unfamiliar with VAs and have difficulties understanding the technology, the interviewers played a series of videos demonstrating the functions of VAs (such as setting reminders, searching for information, controlling home appliances, etc.). Additionally, the interviewers used Tmall Genie Cube Sugar (a widely used smart speaker in China with no secondary modality) for the demonstration. Each elderly participant engaged in a conversation with the smart speaker to gain an understanding of VAs. The interviewer provided explanations to participants if they had any areas of unfamiliarity. Once all participants clearly understood the functionalities of VAs, the interviews began. The interviews were concluded when the information obtained from the participants reached saturation (Braun and Clarke, 2006).

The interview outline consisted of three aspects:

1. What do you think of VAs?
2. Will you use VAs in the future?
3. What factors influence older adults’ intentions to use VAs (product characteristics and personal characteristics)?

All interviews were audio-recorded and transcribed. To ensure the accuracy and consistency of the transcribed text with the interview results, researchers used the recordings to verify the transcribed results.

### 5.3 Data analysis

The interview data were analyzed using thematic analysis, following open, axial, and selective coding (Braun and Clarke, 2006). The first author continuously discussed the emerging themes with another author until the data reached saturation with repeated themes, and no new information emerged.

1. Open coding: The textual data was broken down into meaningful units (words, phrases, or sentences), and these dissected units were categorized and classified to form initial codes.
2. Axial coding: The codes obtained from open coding were further refined, adjusted, and grouped based on their similarities or connections. Similar or related codes were merged, and the underlying relationships between codes were clarified and organized through constant comparison, resulting in the development of categories.
3. Selective coding: At a more abstract level, the data from axial coding were further processed. The developed categories were consolidated and interconnected to explain the overarching themes and provide a comprehensive understanding of the data.

Through this rigorous analysis, the thematic analysis allowed for a systematic exploration and interpretation of the interview data.

### 5.4 Results

This research encompasses 121 reference points (i.e., meaningful units) extracted to obtain 14 relatively independent initial concepts. Subsequently, centered around the core concept of factors influencing older adults’ acceptance of VAs, the 14 initial concepts were compared and categorized into seven distinct categories. Table 1 displays the initial concepts obtained through open coding and the categories formed by axial coding. Due to space constraints, only one reference point for each initial concept was shown here.

**Table 1:** Open coding and categorization.

Category	Original data	Initial codes [reference points]
Perceived privacy and security risks	5: I’m concerned about voice assistants stealing information about my financial accounts as well as my assets.	Worrying about security risks [2]
	7: The risk is okay; I also have no privacy.	Ignoring risks [6]
No or low confidence in using smart voice products	2: I do not want to use VAs because the senior citizens are too old to learn intelligent products.	Older adults lack confidence to utilize smart device. [10]
	6: I received guidance from others, but after a period of time, I would forget, and despite their attempts to teach me, I still could not grasp it.	Learning smart products with the assistance of others can also be quite challenging [8]
Perceived limited value compared to other mediums	8: Because now functions of VAs can be dispensable for the elderly.	Functionality is dispensable [8]
	11: The information from iPad is enough; the smart speaker is just a speaker.	There are other ways to substitute [15]
Perceived benefits for supporting independent living for older adults	5: VAs can help me find the phone, play the weather forecast, and sing a piece of opera for me; quite convenient.	The features such as companionship, entertainment, and living support are suitable for the elderly [13]
	17: How convenient and simple VAs are, very convenient for the elderly.	Voice assistants are suitable for the elderly [9]
Easy to use overall, but older adults still have difficulty using it	19: Tmall Genie is relatively simple to use; it will play what I want to listen.	Perceive that the process of using voice assistants is simple [14]
	7: We still have problems speaking Mandarin. VAs did not understand. I just said 11:20. VAs heard it as 2:00. Recognizing accent correctly is very important.	Troubled by the structured conversations and dialects that cannot be recognized [9]
Seeking comfort and stability, resisting change	10: Our generation finds it challenging to embrace new things and often tends to stick with established life habits.	Older adults tend to maintain the status quo [12]
	12: In my daily work and life, I tend to be more conservative, and I gradually accept many things over time.	Resist changes in life [7]
Perceived enjoyable	7: The process of using voice assistant makes me feel quite happy.	Content with the voice assistants experience
	18: I like to use simple products. The use of VAs makes me happy.	Easy to use makes the older adults feel happy [3]

**Figure 3:**
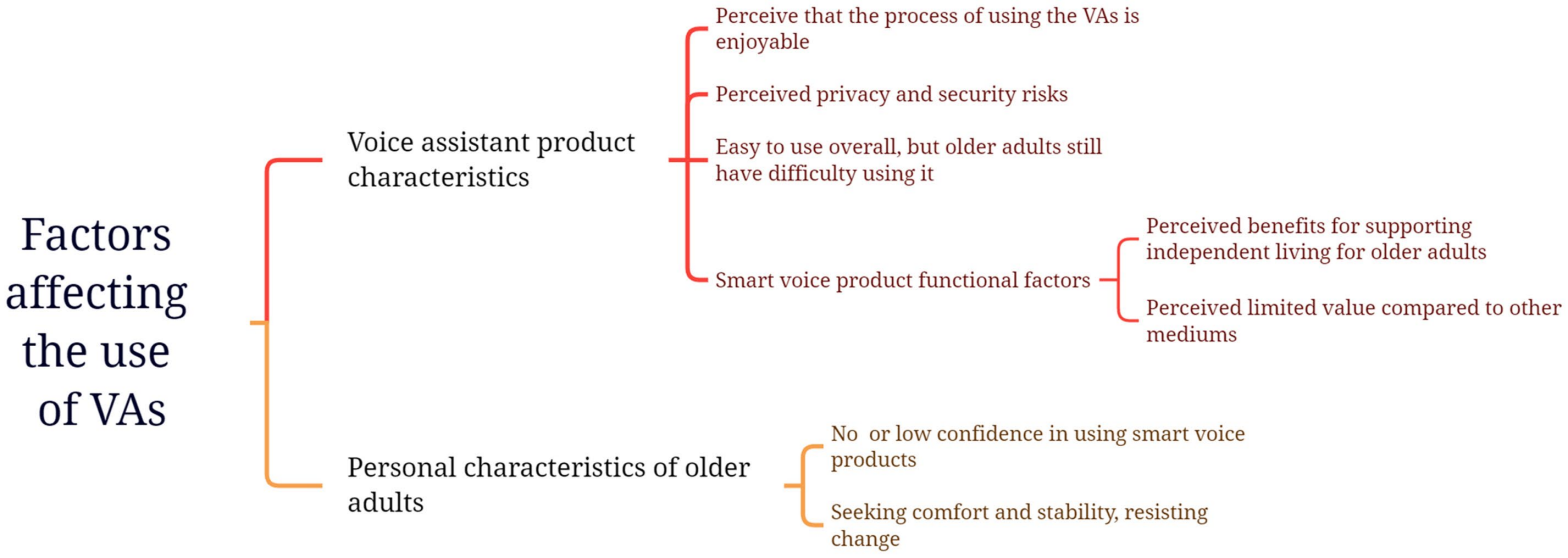
Categories influencing older adults’ acceptance of VAs.

Finally, this study consolidates the developed categories and constructs that influence older adults’ acceptance of VAs into two themes: personal characteristics and VA product characteristics. These themes and connected categories were illustrated in Figure 3.

### 5.5 Summary and discussion

The results of the semi-structured interviews revealed factors influencing the usage of VAs among older adults can be categorized into two aspects: VA product characteristics and personal characteristics of older adults. We aligned the themes identified through thematic analysis with existing variables, ultimately determined that VA product characteristics including perceived usefulness, perceived ease of use, perceived enjoyment, value barrier, and perceived trust, while the personal characteristics including technological self-efficacy and dispositional resistance to change.

#### 5.5.1 Product characteristics that could influence the usage of VAs among old adults

Regarding VA product characteristics, the interviews revealed that most older adults exhibited a positive attitude towards VAs, considering them as natural and straightforward interactions that support daily living and enable older adults to live more independently. The ease of activation through wake-up words or physical buttons on the devices makes VAs accessible to older adults, lowering the barriers to their usage. These results indicated perceived usefulness and ease of use were common factors influencing older adults’ acceptance of VAs, which aligns with previous research findings (Song et al., 2022; Werner et al., 2023). However, some barriers affected the perceived usefulness and ease of use for older adults. For instance, short listening times of VAs, structured question-and-answer approaches, and limitations in speech recognition quality for users with accents could hinder the perceived usability. Additionally, many participants tended to compare VAs with other means of assistance, sometimes perceiving their value as optional. This is consistent with findings from Trajkova and Martin-Hammond (2020), who observed a high churn rate of VAs among older adults due to the lack of perceived utility. These results support hypotheses H1, H2, H5, and H6.

Similarly, consistent with the findings of Kim (2021), the interviews also demonstrated that older adults had concerns about privacy and security risks related to VAs. These concerns were associated with the continuous listening capability of VAs in private spaces like homes. Furthermore, participants worried that others could easily access their personal information by conversing with VAs. Among these risks, financial security was perceived as a more profound concern for some old adults when using VAs. However, some users believed that the security risks posed by VAs could be disregarded. One reason for this belief was cognitive bias, where they were unaware of the risks of using VAs. Additionally, some users were not concerned about the risks due to their usage patterns, as they believed they would not engage in conversations that involved sensitive information. This finding partially supports hypothesis H7.

On the other hand, the interviews revealed that some older adults expressed pleasure and delight in using VAs due to their simplicity, which aligns with previous research findings. Akdim et al. (2022) found that when users perceive virtual communities have humanized interfaces, the perceived enjoyment is enhanced, which could encourage continued engagement. Emotional needs continue to grow with aging. The interaction of VAs, which are naturally simple and create a relaxed atmosphere, meet the emotional needs of older adults and may influence perceived usefulness. Therefore, we add the following hypotheses:

*H10*: Perceived ease of use positively influences perceived enjoyment.

*H11*: Perceived enjoyment positively influences perceived usefulness.

#### 5.5.2 Personal characteristics that could influence the usage of VAs among old adults

Regarding the personal characteristics of older adults, the interviews revealed that the belief in their ability to use VAs successfully may directly influence their usage behavior. If older adults perceive themselves as lacking the capability to use VAs, they might be more inclined to give up on using them. Werner et al. (2023) also found that older adults who willingly use VAs likely have a higher level of confidence in their ability to interact successfully with VAs, while those who do not use VAs may lack the same level of confidence. This finding supports hypothesis H9.

Furthermore, the interviews revealed that older adults tend to find contentment in their existing comfort or stability and are often hesitant to explore unfamiliar environments or adopt new technologies. Many older adults cited this as a reason for their refusal to use VAs. It is inherent in human nature to cling to established habits rather than embracing change and venturing into unknown territories (Mazar and Wood, 2022). In extensive research on resistance to change and individual differences, Oreg (2003) proposed that this pursuit of comfort, stability, and resistance to change is not merely a situational behavior but a fundamental personality trait. Some scholars have emphasized the significance of the DRTC in technology adoption, but its impact has not been thoroughly addressed in IT adoption literature, necessitating further investigation (Mzoughi and M’Sallem, 2013; Touchaei and Hashim, 2023). Currently, DRTC has not received attention in the context of VAs. This study considers DRTC a potential barrier for older adults in adopting VAs, as it can influence their value assessment and usage intention towards VAs. Therefore, we add the following hypotheses:

*H12*: DRTC positively influences the value barrier.

*H13*: DRTC negatively influences behavioral intention.

**Figure 4:**
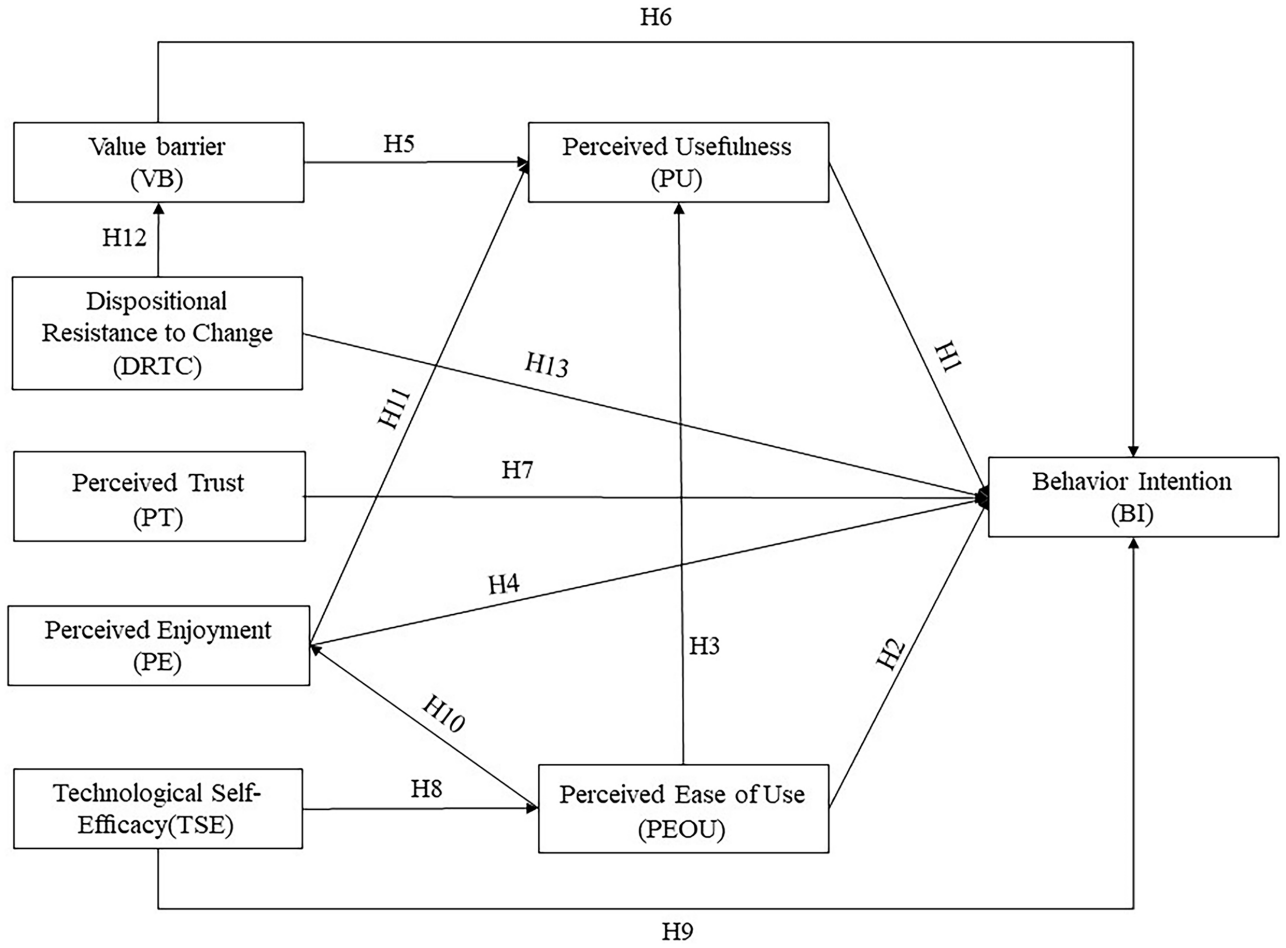
The final hypothesis model of current study.

Our research has yielded valuable insights that were previously overlooked by using semi-structured interviews. One such example is the ease of use that typifies VAs. The interviews revealed that ease of use can be a source of pleasure for older individuals and also enhances their perception of the usefulness of VAs. Furthermore, our findings indicated that a predisposition to resist innovation can impact the acceptance of VAs among older adults. Consequently, we have incorporated hypotheses H10 to H13 into the original model, resulting in the final hypothesis model depicted in Figure 4.

## 6 Study 2: questionnaire survey

To test the proposed model for older adults’ acceptance of VAs, a survey was designed and conducted. Data analyses were conducted using partial least squares structural equation modeling (PLS-SEM).

### 6.1 Participants

The selection criteria and participant recruitment process were consistent with Study 1. A total of 154 individuals aged 60 and above were recruited. Among them, 153 participants (Mean age = 70.08, SD = 5.19, 60.1% female) were included in the study. The characteristics of the participants are shown in Table 2. Participants’ experience with VAs can be found in Table 3.

**Table 2:** Characteristics of participants (*N* = 153).

Characteristics	Item	Frequency	Percentage
Gender	Male	61	39.90
	Female	92	60.10
Age	60–64	20	13.10
	65–69	51	33.30
	70–74	52	34.00
	≥75	30	19.60
Living arrangement	With family member(s)	127	83.00
	Living alone	26	17.00
Marital status	Married	129	84.30
	Divorced/separated	16	10.50
	Widowed	6	3.90
	Never married	2	1.30
Education level	Elementary	4	2.60
	Middle school	41	26.80
	High school	42	27.50
	University and above	66	43.10
Income	<2000 yuan	4	2.60
	2000–4,000 yuan	59	38.60
	4,000–6,000 yuan	37	24.20
	6,000–8,000 yuan	34	22.20
	8,000–10,000 yuan	15	9.80
	>10,000 yuan	4	2.60
Smart voice assistant usage frequency	Non-user	99	64.70
	Occasional user	26	17.00
	General user	12	7.80
	Frequent user	10	6.50
	Very frequent user	6	3.90

**Table 3:** Frequency of specific use of participants with voice assistants using experience (*N* = 54).

Specific use	Frequency	Percentage
Listen to music, opera, radio, etc.	54	100
Checking the weather	26	48.10
Daily conversation	19	35.20
Set reminders	18	33.30
Search for information	14	25.90
Control home appliances	13	24.10
Find phone	7	12.90
Other	3	5.60

### 6.2 Questionnaire survey methodology

#### 6.2.1 Research design

We employed a quantitative approach using structured questionnaires to investigate the impact factors of older adults’ intentions to use VAs. The questionnaire completion process involved face-to-face interviews to facilitate synchronous communication between the interviewer and the older adults while completing the questionnaire. The accuracy of responses was verified by repeating the participants’ answers, which took approximately 30 min each participant. To ensure consistency in the survey, the interviews were conducted by the same experienced researcher. The questionnaire data was entered by two well-trained researchers and cross-checked upon completion.

#### 6.2.2 Measurement development

The survey questionnaire for this study included the variables related to acceptance of VAs and demographic information. Table 4 presents the items related to the acceptance of VAs and its’ impact factors used in the current study. All the variables and indicators in the questionnaire were measured by a seven-point Likert scale ranging from 1 (strongly disagree) to 7 (strongly agree).

**Table 4:** Constructs and measurements.

Construct	Measurement item		References
Behavior Intention (BI)	BI1	I am interested in using VAs	Davis (1989); Li et al. (2019)
	BI2	Using VAs is a good idea.	
	BI3	I intend to use VAs in the future.	
Perceived Enjoyment (PE)	PE1	I think VAs are attractive	Nguyen et al. (2018)
	PE2	I think it is fun to use VAs.	
	PE3	I find using VAs enjoyable.	
Perceived Ease of Use (PEOU)	PEOU1	I think it is relatively easy to use VAs	Chen and Chan (2014); Davis (1989)
	PEOU2	I feel that I can use the VAs skillfully.	
	PEOU3	I feel that the process of interacting with the VAs is straightforward and easy to understand	
Perceived Usefulness (PU)	PU1	I feel that the VAs can provide me with help	Chen and Chan (2014); Davis (1989)
	PU2	I feel that using VAs makes life more convenient.	
	PU3	I think VAs are very useful in life.	
	PU4	I think using VAs can make my life easier.	
Technological Self-Efficacy (TSE)	TSE1	I think I can master using VAs by learning it myself	Chen and Chan (2014)
	TSE2	I think I can complete the operation of VAs with the help of others.	
	TSE3	By reading the manual, I feel confident about using VAs.	
	TSE4	I can learn to use VAs if I put in the effort.	
Value barrier (VB)	VB1	VAs do not have significant advantages over other mediums	Lee and Kim (2022); Trajkova and Martin-Hammond (2020)
	VB2	It was difficult to find uses for VAs that were essential to daily.	
	VB3	It is more convenient to get information through other mediums rather than VAs.	
	VB4	If I can choose other mediums, I will use other mediums to do the same thing.	
Perceived Trust (TR)	TR1	I believe my information is safe when I use VAs	Nguyen et al. (2018)
	TR2	I do not think there is a privacy risk when using VAs.	
	TR3	I do not think I will be fooled by using VAs.	
Dispositional Resistance to Change (DRTC)	DRTC1	I generally consider changes to be a negative thing.	Oreg (2003)
	DRTC2	When things do not go according to plan, it stresses me out.	
	DRTC3	I sometimes find myself avoiding changes that I know will benefit me.	
	DRTC4	I do not change my mind easily.	

#### 6.2.3 Procedures

Given the potential unfamiliarity of the elderly with VAs, an introductory session was conducted before participants completed the questionnaire, following a similar approach as in Study 1.Older adults who completed the experiment were rewarded with a 30 yuan monetary incentive.

### 6.3 Data analysis

This study used the PLS-SEM approach for data analysis. The measurement model and structural model were analyzed using Smart PLS 3.3.9. PLS-SEM is selected due to its minimal sample size requirements, which are determined by the greater of the following two criteria: (1) ten times the maximum number of formative indicators employed to measure a single construct, or (2) ten times the maximum number of structural paths directed towards a specific latent construct within the structural model. It is suitable for analyzing complex structural equations with multiple latent variables and related items and is applicable for exploratory and predictive models in research and data analysis (Hair et al., 2017). PLS-SEM is widely used in various social sciences and related disciplines (Hair et al., 2019) and has been employed in TAM studies (Sagnier et al., 2020; Xu et al., 2023). As recommended, the data analysis was conducted in two stages. Firstly, we assessed the internal consistency, the convergent validity, and the discriminant validity of the measurement model (Hair et al., 2021). While it is unnecessary to report fit metrics, we reported the Standardized Root Mean Square Residual (SRMR), which is considered useful in detecting model misspecification (Xu et al., 2023). Secondly, following satisfactory results in the first stage, we proceeded with the structural model to test our hypotheses.

### 6.4 Results

#### 6.4.1 Measurement model assessment

Composite Reliability (CR) and Cronbach’s alpha assessed the internal consistency reliability. The combined performance of these two indicators objectively determines the internal quality of the constructed measurement model. Following the standards proposed by Hair et al. (2011), the minimum threshold for both indicators is set at 0.7. As shown in Table 5, all CR values are above 0.8, and all Cronbach’s alpha values are above 0.7.

**Table 5:** Convergent validity and construct reliability of measures.

Constructs	Items	Factor loading	Cronbach’s alpha	CR	AVE
Behavior Intention (BI)	BI1	0.949	0.933	0.957	0.882
	BI2	0.947			
	BI3	0.921			
Perceived Enjoyment (PE)	PE1	0.924	0.936	0.959	0.886
	PE2	0.952			
	PE3	0.949			
Perceived Ease of Use (PEOU)	PEOU1	0.877	0.780	0.872	0.695
	PEOU2	0.824			
	PEOU3	0.798			
Perceived Usefulness (PU)	PU1	0.906	0.930	0.950	0.827
	PU2	0.906			
	PU3	0.897			
	PU4	0.928			
Technological Self-Efficacy (SE)	TSE1	0.887	0.908	0.935	0.784
	TSE2	0.865			
	TSE3	0.900			
	TSE4	0.888			
Value barrier (VB)	VB1	0.803	0.839	0.891	0.673
	VB2	0.802			
	VB3	0.816			
	VB4	0.859			
Dispositional Resistance to Change (DRTC)	DRTC1	0.814	0.893	0.926	0.759
	DRTC2	0.857			
	DRTC3	0.916			
	DRTC4	0.894			
Perceived Trust (PT)	TR1	0.918	0.866	0.918	0.788
	TR2	0.882			
	TR3	0.863			

Convergent validity was evaluated by average variance extracted (AVE) and items outer loading. All latent variables, each consisting of three or more items, have standardized factor loadings greater than 0.7, and the AVE values for all latent variables are above 0.5, indicating high convergent validity of the measurement items in this study (Hair et al., 2021).

To assess discriminant validity, we used the Fornell-Larcker criterion and the HTMT values. Based on the Fornell-Larcker criterion, the bold values on the diagonal represent the square root of the AVE for each latent variable, and the values in the lower half of the diagonal represent the correlations between each latent variable and other latent variables. In this case, all AVE values were more significant than correlations with other constructs, and the HTMT values were less than 0.85 for all the constructs, confirming that the model has satisfactory discriminant validity (Hair et al., 2021). Table 5 presents the factor loadings and AVE values for all the constructs. Tables 6, 7 provide the AVE square root on the diagonal and the correlations among constructs and the HTMT results, respectively.

**Table 6:** Correlation matrix among constructs and square root of AVEs.

	BI	PE	DRTC	PEOU	PU	TSE	PT	VB
BI	**0.939**							
PE	0.658	**0.942**						
DRTC	−0.540	−0.537	**0.871**					
PEOU	0.510	0.455	−0.361	**0.834**				
PU	0.597	0.479	−0.332	0.395	**0.909**			
TSE	0.670	0.499	−0.415	0.488	0.436	**0.885**		
PT	0.458	0.378	−0.322	0.283	0.284	0.375	**0.888**	
VB	−0.403	−0.442	0.347	−0.275	−0.351	−0.255	−0.297	**0.820**

BI, behavior intention; PE, perceived enjoyment; DRTC, dispositional resistance to change; PEOU, perceived ease of use; PU, perceived usefulness; TSE, technological self-efficacy; PT, perceived trust; VB, value barrier. Diagonal values in boldface are the square roots of the AVEs.

**Table 7:** HTMT (heterotrait-monotrait ratio of correlations) results.

	BI	PE	DRTC	PEOU	PU	TSE	PT	VB
BI								
PE	0.701							
DRTC	0.593	0.586						
PEOU	0.593	0.525	0.431					
PU	0.637	0.513	0.365	0.459				
TSE	0.726	0.540	0.461	0.580	0.472			
PT	0.505	0.414	0.363	0.331	0.306	0.417		
VB	0.445	0.489	0.397	0.324	0.380	0.294	0.342	

BI, behavior intention; PE, perceived enjoyment; DRTC, dispositional resistance to change; PEOU, perceived ease of use; PU, perceived usefulness; TSE, technological self-efficacy; PT, perceived trust; VB, value barrier.

Moreover, the estimated SRMR value for the model in this study is 0.06, which is below the threshold of 0.08 (Hu and Bentler, 1999; Kline, 2023). This indicates that the model is acceptable in terms of model fit.

#### 6.4.2 Structural model assessment

Before assessing the inner structural model, common method bias, each construct’s multicollinearity test, model fit evaluation, and descriptive statistics were calculated. To examine whether common method bias was present in our data, we employed Harman’s single-factor test. The highest eigenvalue corresponded to the first component, which accounted for 0.396 of the variances. This falls below the established threshold of 0.4 (Podsakoff et al., 2003), indicating that common method bias was not a significant concern in our study. Multicollinearity was assessed using variance inflation factors (VIF). The results showed that VIF values of all constructs ranged from 1.000 to 1.944, well below the threshold of 5.0 (Hair et al., 2016), indicating the absence of multicollinearity.

We used bootstrapping to test the relationships hypothesized in our model. Path significance was tested using a bootstrapping technique for the 153 cases with 5,000 samples (Hair et al., 2011). Table 8 lists all path coefficients and their significance. To assess the predictive strength of the model, we reported R^2^ values for each endogenous variable. As a rule of thumb, we followed (Hair et al., 2011) to report the R^2^ values where R^2^ of 0.25, 0.50, and 0.75 are considered weak, moderate, and substantial, respectively. Results suggest that our model can explain 67.4% of the variance in behavioral intention (moderate), 27.3% of the variance in perceived usefulness (moderate), 23.8% of the variance in perceived ease of use (weak), 20.7% of the variance in perceived enjoyment (weak), and 12.0% of the variance in value barrier (weak). These R2 values are comparable to those reported in the literature (Xu et al., 2023).

**Table 8:** Results of path analysis and hypotheses testing.

Hypotheses		Path coefficient	*T*	*p*	Result
H1	PU → BI	0.233**	2.720	0.007	Supported
H2	PEOU → BI	0.071	1.048	0.295	Not supported
H3	PEOU → PU	0.208*	2.544	0.011	Supported
H4	PE → BI	0.224**	2.977	0.003	Supported
H5	VB → PU	−0.154*	2.177	0.029	Supported
H6	VB → BI	−0.042	0.694	0.488	Not supported
H7	PT → BI	0.114	1.785	0.074	Not supported
H8	TSE → PEOU	0.488***	5.566	<0.001	Supported
H9	TSE → BI	0.311***	3.672	<0.001	Supported
H10	PEOU → PE	0.455***	6.644	<0.001	Supported
H11	PE → PU	0.316**	2.829	0.005	Supported
H12	DRTC → VB	0.347***	4.617	<0.001	Supported
H13	DRTC → BI	−0.136*	2.015	0.044	Supported

BI, behavior intention; PE, perceived enjoyment; DRTC, dispositional resistance to change; PEOU, perceived ease of use; PU, perceived usefulness; TSE, technological self-efficacy; PT, perceived trust; VB, value barrier.

^*^p < 0.05; ^**^p < 0.05; ^***^p < 0.001.

**Figure 5:**
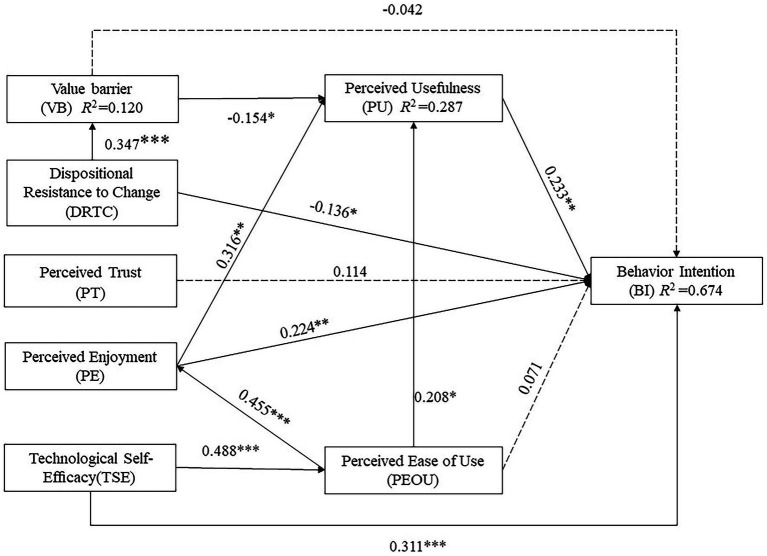
Results of the structural model of current study Note: *p < 0.05, **p < 0.05, ***p < 0.001; Three dotted lines indicating non-significant paths were added in making all proposed factors shown in an integral model.

According to the path analysis, perceived usefulness, perceived enjoyment, and technological self-efficacy positively influenced behavioral intention. DRTC had a negative impact on behavioral intention. However, the effects of perceived ease of use, value barrier, and perceived trust on behavioral intention were not significant. This means that H1, H4, H9, and H13 are supported, while H2, H6, and H7 are not supported. Furthermore, perceived ease of use had a positive influence on perceived usefulness and perceived enjoyment, while perceived enjoyment had a positive influence on perceived usefulness. This means that H3, H10, and H11 are supported. Lastly, technological self-efficacy had a positive influence on perceived ease of use, DRTC had a positive influence on value barrier, while value barrier had a negative impact on perceived usefulness. This means that H5, H8, and H12 are supported. Table 8 and Figure 5 present the results of the hypothesis testing and the structural model, respectively.

## 7 Conclusion and discussion

This study categorized the factors influencing the acceptance of VAs among older adults into VAs product characteristics and personal characteristics of older adults. Consistent with previous research, it was observed that perceived usefulness, perceived enjoyment, and technological self-efficacy significantly affected behavioral intention (Nguyen et al., 2018; Song et al., 2022; Zhong et al., 2022). However, there were several differences between the model created and the results produced by existing research. Primarily, inconsistencies existed in the research findings. Perceived trust and perceived ease of use were previously identified as significant factors influencing older adults’ acceptance of VAs (Kong and Woods, 2018; Kebede et al., 2022; Song et al., 2022). However, our study did not find evidence supporting this. Nonetheless, our model indicated how ease of use influenced the mechanism of older adults’ acceptance of VAs. Secondly, our research identified other important factors that influenced older adults’ acceptance of voice assistants, such as DRTC, which was not previously highlighted as a significant factor affecting older adults’ acceptance of VAs. The following sections explain the results in detail.

### 7.1 VAs product characteristics

#### 7.1.1 Perceived usefulness, perceived ease of use, and perceived enjoyment

The result showed that perceived usefulness positively influenced behavioral intention, consistent with previous studies on the acceptance of VAs (Song et al., 2022; Zhong et al., 2022), indicating that older adults who perceived the benefits of VAs are more likely to have a higher intention to adopt them. Consequently, older adults may be inclined to embrace VAs due to their perception of VAs as valuable and convenient technologies. Furthermore, results also showed a positive impact of perceived enjoyment on behavioral intention, which was consistent with previous research on VAs (Nguyen et al., 2018; Zhong et al., 2022). As older adults perceive time as limited, they prioritize emotional satisfaction over other goals (Carstensen, 1995), and positive user emotions and experiences contribute to their acceptance of the product. If users experience perceived enjoyment through their interactions, it can lead to an expectation of internal psychological rewards, encouraging them to continue using the technology (Suki and Suki, 2011; Akdim et al., 2022). Moreover, perceived enjoyment positively influenced perceived usefulness, which may be related to fulfilling their emotional needs through positive user experiences. Schlomann et al. (2021) also found that some older adults view smart speakers as social companions, meeting their emotional needs and enhancing their perceived usefulness.

The impact of perceived ease of use on behavioral intention wasn’t significant, which contradicts previous studies (Song et al., 2022). However, perceived ease of use had a direct influence on perceived usefulness and perceived enjoyment. This may be because the effect of perceived ease of use on behavioral intention was indirect. In other words, if older adults perceive the natural and simplistic nature of voice interaction, they would consider VAs useful and enjoyable, leading to the intention to use them. VAs possess the distinctive advantage of being easy to use, allowing older adults to use them effectively without any major operational difficulties. While it is not considered a direct factor that influences the intention to use, it does play a significant role in enhancing the user experience including the perceived usefulness and enjoyment. This indicated the crucial role of perceived usefulness and perceived enjoyment in the process of older adults accepting VAs.

#### 7.1.2 Value barrier

Results indicated that value barrier did not directly influence behavioral intention, suggesting that VAs were not perceived as hindrances. This is not considered a barrier because VAs are not complicated and have a humanized interface. However, value barrier negatively impacted perceived usefulness, indicating that if older adults perceived VAs as less valuable compared to existing methods, they might have had doubts about their practicality. The role of value in motivating older adults to adopt new technologies was crucial (Melenhorst et al., 2006). Older adults need to have gained more benefits than the effort required to understand and use VAs to perceive no value barriers.

#### 7.1.3 Perceived trust

The research indicated that perceived trust did not directly influence behavioral intention, but numerous studies found that fear of safety and invasion of privacy were barriers to digital engagement (Fischl et al., 2017; Kong and Woods, 2018; Zambianchi et al., 2019; Kebede et al., 2022). However, privacy calculus theory posits that disclosing personal information is based on a trade-off between perceived privacy risks and perceived benefits of information disclosure (Dinev and Hart, 2006). If the perceived benefits of information disclosure outweigh the perceived risks, users may be willing to disclose personal information despite their privacy concerns. Schomakers and Ziefle (2022) suggested that as long as certain boundaries are not crossed, security-related benefits may outweigh privacy concerns. For older adults, although they are aware that using VAs may lead to a partial loss of privacy due to their real-time listening capabilities, they may still choose to use them due to other features, such as simple and enjoyable interactions, after weighing the trade-offs. Additionally, the COVID-19 pandemic has increased social isolation among older adults, impacting their sense of security at home and their need for support, particularly for those living alone, which may lead them to be more willing to disclose some information to enhance their sense of security (Conroy et al., 2020; Schomakers and Ziefle, 2022).

### 7.2 Personal characteristics of older adults

#### 7.2.1 Technological self-efficacy

The research findings indicated that technological self-efficacy had a significantly positive impact on perceived ease of use, consistent with previous studies on VAs (Song et al., 2022). Moreover, technological self-efficacy also significantly positively influenced behavioral intention, and it was the strongest predictor of older adults’ intention to use VAs, suggesting older adults’ perception of their capabilities plays a substantial role in the acceptance of VAs. Although this contradicted the conclusions of TAM, which indicated that self-efficacy was not considered as a predictor of behavioral intention (Venkatesh and Bala, 2008), TAM’s samples mainly consisted of young individuals, who generally possessed higher levels of self-efficacy in using technology (Czaja et al., 2006). Recent research suggested that older adults’ technology usage was more driven by their perception of their abilities than the technological features represented in TAM (Jokisch et al., 2021). This could be attributed to cohort effects, as the current generation of adults has already acquired basic information and communication technology skills daily, leading to a generally higher rate of technology adoption (Charness and Boot, 2022). They have more role models in their social environment who demonstrate beneficial integration of the internet into daily life or provide support in case of technological issues. However, these advantages may not be as prevalent among older adults. As a result, technological self-efficacy emerged as the most influential factor in predicting the acceptance of VAs among older adults, surpassing the significance of perceived usefulness.

#### 7.2.2 Dispositional resistance to change (DRTC)

The result revealed the negative impact of DRTC on behavioral intention, indicating the inhibiting effect of DRTC on seniors’ intention to adopt VAs. Devaraj et al. (2008) also suggested that incorporating personality traits into theoretical models can enhance their predictive ability in explaining technology adoption by users. The result of this study could partly explain why some seniors show reluctance in VAs adoption. Since VAs are regarded as an innovation, they may be incompatible with their habits and of little practical use. Furthermore, older adults exhibit a high level of resistance to change and technological innovation (Hoque and Sorwar, 2017), and the tendency to embrace familiarity impedes their experience of VAs.

In addition, DRTC positively influenced value barrier, indicating that older adults with a high inclination towards resisting innovation tend to perceive VAs as less valuable than existing products. This may be because VAs disrupt their traditional way of life. Individuals accustomed to and satisfied with their conventional lifestyle may alter their value assessment of VAs to maintain the status quo and persuade themselves (Talukder et al., 2020).

## 8 Implications

Over the past decade, VAs have become widespread and integrated into various devices used in various scenarios. The benefits of VAs should be accessible to everyone, especially vulnerable groups like older adults (Song et al., 2022). Initially designed for younger users, research on VAs has paid less attention to the personal characteristics of older adults. However, due to the heterogeneity of their physiological and psychological functions, older adults differ significantly from younger individuals regarding technology acceptance (Kim and Choudhury, 2021). This study identified factors influencing the acceptance of VAs among older adults, contributing to the development of enhanced voice assistants to facilitate the adoption of smart technology among seniors. Moreover, previous research has indicated that VAs support older adults in various domains. They assist in daily health activities, such as health monitoring, medication management, and meal planning, thereby enhancing overall well-being. VAs also serve as social companions to some extent, addressing social isolation and loneliness. By identifying factors influencing older adults’ acceptance of VAs, this study sheds light on the social issues faced by this demographic, including social isolation and loneliness, health and well-being concerns, ageism and stereotypes, as well as technology accessibility.

Regarding VA product features, the critical focus of improvement and optimization should be enhancing speech recognition and natural language processing quality. The perceived practicality, ease of use, and enjoyment of VAs depend on speech recognition accuracy and natural language processing capabilities. Specifically, efforts can be made to reduce the impact of environmental noise and user accents on speech recognition. Providing appropriate feedback, such as offering reasons for query failures or suggestions to improve query results, can guide users correctly and enhance the responsiveness of VAs.

Concerning the negative impact of value barrier on perceived usefulness, it is worth noting that smart voice products, such as smart speakers, are not specifically designed for older users, and many of the functions they need may not be available. Therefore, adding more features suitable for older adults, such as fall detection alerts, medication purchases, and hospital appointment arrangements, can enhance older adults’ perception of usefulness.

Furthermore, concerning the positive impact of perceived enjoyment on the use of VAs among older adults, it is essential to consider the prevalent risk of social isolation and susceptibility to feelings of loneliness and depression faced by this vulnerable demographic. Designers should contemplate crafting a unique persona for VAs, which can significantly reduce the psychological distance perceived by older users. Meticulous consideration should be given to creating an ideal personality, encompassing gender, tone, and speaking style. Given that older adults bear a lower cognitive load, employing easily memorable and concise vocabulary, such as “OK” and “got it,” can be beneficial (Song et al., 2022). Designers can also endeavor to break free from passive reliance on users’ established patterns and actively engage with them. Initiating greetings and informal conversations or increasing interaction during tasks can make the user experience more enjoyable and engaging.

Although trust does not directly influence the acceptance of VAs among older adults, it does not diminish the importance of trust-related concerns to them. Trust considerations may very well be a part of the deliberation process for older individuals. Given that voice data constitutes a common and significant category of information in people’s daily lives, designers should prioritize enhancing user data storage and utilization transparency. Providing clear information on how users can access and delete their voice data and offering privacy protection clauses can foster a sense of trust and reassurance among older users.

Regarding the personal characteristics of older adults, this study further reveals that individuals with higher levels of technological self-efficacy demonstrate greater ease of use and willingness to use VAs. When promoting VAs, marketers should adopt different communication strategies for older adults with varying levels of self-efficacy. For those with lower self-efficacy, it is essential to emphasize the ease of use of VAs, particularly highlighting the advantages over traditional interaction methods.

Considering the negative impact of DRTC on behavioral intention, personalized services tailored to their preferences could be implemented. VAs could be designed to learn and adapt to users’ interactions in real-time, identifying recurring usage patterns and adapting to individual habits. Allowing users to customize voice output settings, such as speech speed, tone, and intensity, can accommodate the lifestyle preferences of older adults and reduce the influence of DRTC. Furthermore, VAs should possess the capability to operate a wide array of devices, including smartphones, televisions, computers, and such. This expansion of usage scenarios for various applications facilitates the seamless integration of intelligent services into users’ lives (Abdolrahmani et al., 2018; Esau et al., 2022), thereby attenuating the impact of DRTC.

## Data availability statement

The raw data supporting the conclusions of this article will be made available by the authors, without undue reservation.

## Ethics statement

Ethical review and approval was not required for the study on human participants in accordance with the local legislation and institutional requirements. All participants signed an informed consent form.

## Author contributions

XC: Conceptualization, Data curation, Formal analysis, Investigation, Methodology, Project administration, Resources, Software, Supervision, Validation, Writing – original draft, Writing – review & editing. HZ: Conceptualization, Data curation, Formal analysis, Investigation, Methodology, Validation, Writing – original draft, Writing – review & editing. BZ: Conceptualization, Data curation, Formal analysis, Methodology, Validation, Writing – review & editing, Investigation. DW: Conceptualization, Project administration, Resources, Supervision, Writing – review & editing, Methodology. CC: Conceptualization, Data curation, Investigation, Writing – review & editing. XB: Conceptualization, Project administration, Resources, Supervision, Writing – original draft, Writing – review & editing, Methodology.
